# Malaria in children of Tshimbulu (Western Kasai, Democratic Republic of the Congo): epidemiological data and accuracy of diagnostic assays applied in a limited resource setting

**DOI:** 10.1186/s12936-016-1142-8

**Published:** 2016-02-11

**Authors:** Simona Gabrielli, Livia Bellina, Giovanni Luigi Milardi, Boniface Kabasele Katende, Valentina Totino, Valerio Fullin, Gabriella Cancrini

**Affiliations:** Dip. Sanità Pubblica e Malattie infettive, Università “Sapienza”, P.le Aldo Moro 5, 00185 Rome, Italy; MobileDiagnosis Onlus, via Sciuti 180, 90144 Palermo, Italy; St François Hospital, Tshimbulu, BP 185, Kananga, Western Kasai Democratic Republic of Congo

**Keywords:** *Plasmodium falciparum*, *Plasmodium malariae*, *Plasmodium vivax*, *Plasmodium ovale*, *Babesia microti*, Children, Western Kasai (Democratic Republic of the Congo), Rapid diagnostic test, Microscopy, Molecular diagnostics

## Abstract

**Background:**

The literature data on malaria in Western Kasai, DRC, are limited and inadequate. A recent molecular survey there has detected *Plasmodium ovale* and *Plasmodium malariae* as mixed infections with *Plasmodium falciparum*. In Tshimbulu, Western Kasai, during a humanitarian initiative designed to provide children with free preventive screening and to reduce the local high malaria death rate, accurate species identification was performed, in order to collect unambiguous epidemiological data and to evaluate the reliability of locally applied diagnostics.

**Methods:**

Finger pricks provided fresh blood for microscopic analysis (MA), for rapid diagnostic test (RDT) and for molecular diagnostics (MD). MA and RDT were first performed by the local team and then a re-interpretation of the results (on the same slides and on RDT’s taken pictures) was conducted in Italy, where MD were performed.

**Results:**

The analysis was conducted on 306 children; RDT found 80.9 % as *P. falciparum*-positive (37.4 % as two-band positive, *P. falciparum* single infection). MA identified a further four children as positive to *P. falciparum* and six co-infections with *P. ovale.* The second RDT evaluation confirmed a similar infection rate (78.2 %) but interpreted as two-band positive a significantly higher share of tests (56.8 %). MA confirmed 80.0 % of the children as malaria positive and, in addition to *P. falciparum*, identified *P. malariae* (13.8 %), *P. vivax* (3.4 %) and *P. ovale* (2.4 %), and detected *Babesia microti* in 19 smears. MD confirmed all of the species found (*Babesia microti* included), classified as mono-infection with *P. falciparum* a rate of spots comparable to MA revision, and identified all *P. ovale* as *Plasmodium ovale wallikeri*. The RDT used locally proved 93.1 % sensitive and 92.1 % specific for *P. falciparum*.

**Conclusions:**

The malaria prevalence among the children and the presence of four *Plasmodium* species, highlighted in this study, identified a sanitary issue which proved to be more alarming than expected, as it was worsened by the unpredictable presence of *P. vivax* and *Babesia microti* (never before reported in DRC). Each diagnostic tool showed its point of weakness. Therefore, the most correct approach is by the combined use of different, locally available, diagnostic tools.

## Background

In the Democratic Republic of Congo (DRC) the Ministry of Health reports malaria as the main cause of morbidity and mortality, mainly affecting pregnant women and children under 5 years of age. This disease accounts for more than 40 % of all outpatient visits and for 40 % of deaths in children <5 years of age, as they are often hospitalized when their anaemia is too severe [1].

Despite the high impact of the disease, the epidemiological data are inadequate, as they mostly derive from local studies that do not allow for national estimates. Only the 2007 demographic data and 2013–2014 health surveys showed that 33.5 % of women and 34.1 % of children aged six to 59 months were positive in molecular testing [2, 3].

The species mainly represented were *Plasmodium falciparum* (95 % of infections), followed by *Plasmodium malariae* (4.9 %) and *Plasmodium ovale* (0.6 %) [4]. Appropriate laboratory tools are critical for a prompt diagnosis and treatment of the infection, especially in light of the increasing detection of resistance to drugs in many areas of the world [5, 6].

Existing diagnostics include microscopic analysis (MA) of blood (stained in thin and thick films), immunological rapid diagnostic test (RDT) and molecular diagnostics (MD). MA represents the ‘gold standard’ for malaria identification and species determination in many endemic areas as it is relatively inexpensive, locally accessible and the only diagnostic providing parasite counting (parasitaemia). However, several challenges exist in performing MA for routine clinical use, especially in endemic countries where urgency is pressing, infrastructures may be obsolete, and resources are often limited, such as availability of well-trained microscopists. In this scenario misdiagnosis due to low parasitaemia or mixed infections is likely to occur [7]. Additionally, MA may not accurately identify all species [8], as in the case of *Plasmodium knowlesi*, which for a long time was falsely diagnosed as *P. malariae* and later correctly identified in humans only through the use of MD [9]. Therefore, especially in limited resource settings, RDT replaces or supports MA, as it rapidly detects parasite antigens and is easy to use.

Good quality RDT is generally as sensitive as MA in detecting *P. falciparum* (about 100 parasites/µL), whereas it is much less sensitive in identifying other species, and does not determine the parasitic burden [10]. Moreover, RDT can result in false positive up to one month after clearance of parasites, and interference due to presence of heterophile antibodies in patients’ samples has been reported [10].

As far as MD is concerned, while being useful to identify parasite species, to detect low parasitaemia [11] and mixed infections [12], it is expensive, requires equipped laboratories with skilled technicians, does not provide immediate results, and therefore is not suitable for routine diagnosis; its appropriate use is restricted to the research field.

All these diagnostic tools were applied during a two-step malaria survey. The first step was carried out during a humanitarian action conducted by LB who was working with the laboratory team of the Saint François Hospital in the town of Tshimbulu (Western Kasai, rural area of DRC).

In this area of Western Kasai, despite all the therapy and interventions, a high death rate both from anaemia and malaria, diagnosed in a limited resource setting, is still registered. A complete and definitive picture of *Plasmodium* species affecting this population would be necessary and crucial for local public health and epidemiology. To achieve these goals, LB organized a local task force (including three laboratory technicians, two nurses and a local facilitator, together with a volunteer group of mothers) to design and conduct the screening.

The second step was possible due to a scientific, non-profit-making collaboration between the team *in loco* operative and Italian researchers, in order: (1) to submit a large number of children to a preventive screening for malaria, followed by therapy if necessary; (2) to collect detailed epidemiological data, identifying *Plasmodium* species present in Tshimbulu; and (3) to evaluate the appropriateness of assays usually applied to diagnose malaria in local hospitals, where a high number of pyretic and/or anaemic children are often waiting for a diagnosis.

## Methods

### Study area and population

The large town of Tshimbulu (6°29′0″S; 22°51′0″E, Lulua rural district, province of Western Kasai), nestled in the savannah, covers an area of about 10 sq km. The health system in Tshimbulu is part of Dibaya health area, comprising 17 health centres, one hospital of reference and the Saint François Hospital (Catholic Archidiocese of Kananga-owned), which has a catchment area of about 499,000 people. The population lives below subsistence level. There is neither productive activity, nor public energy or water. Householders own insecticide-treated bed nets as the only malaria control measure, but these are often improperly used.

### Study design

In October 2014 the whole community was alerted by local rural radio, organizations and through notice given in churches and markets that a free malaria screening, aimed at preventing the deaths from the infection, was offered to all children under 5 years old. This humanitarian initiative was started in collaboration with the Managing Director and staff of the Saint François Hospital. The hospital is an essential part of the Ministry of Health, which sustained the costs related to diagnostic RDT and drugs, and gave ethical approval. All mothers provided informed consent for their children. Many of them helped by involving more mothers and actively took part in any decision about their children’s treatments, hospitalization and/or therapy administration [artemesinin combination therapy (ACT)] according to national guidelines based on Malaria Operational Plan Fy 2013 (http://www.pmi.gov).

All subjects were examined clinically and axillary temperatures were recorded. Fresh blood was obtained by pricking a child’s finger, and standard malaria thick and thin smears were prepared and stained with 10 % Giemsa solution, following the method described in the WHO Malaria Microscopy Guide (http://apps.who.int). A further 5 µL of blood were used to perform RDT (Standard Diagnostic Inc., Bio Line Malaria Ag Pf/Pan Rapid Test, Cat. No. 05FK60) and, starting from the fifth day, two drops of blood were drawn and used to impregnate a filter paper for MD.

The help of each mother and of a facilitator speaking the local dialect allowed completion of a form about each baby (sex, age, fever attacks, drugs taken, previous malaria attacks), with data on anaemia, fever and course of the infection during therapy.

RDT was locally performed according to manufacturer’s instructions (RDT1), and serially numbered. Each of them was read and then photographed within 15–30 min, all in the same place, position and lighting conditions, controlling the match between each image and the related device test. In this way pictures were available for both a second interpretation (RDT2) and for the final comparison of the findings obtained by means of the three methods.

Giemsa-stained smears were locally examined (triple blinded) by microscopy at 100× in the hospital laboratory (MA1). A whole thick smear was read before declaring a slide negative. Parasite density was estimated by means of the Plus system, and species identification was confirmed on the corresponding thin film. A further (double) blinded microscopic analysis on 290 undamaged available slides was carried out in Italy to evaluate the level of accordance of the results (MA2).

### Molecular analyses

DNA was extracted from a 3 × 3 mm diameter circle of each of the 211 dried blood spots using the commercial kit Dried Blood spot DNA isolation Kit (Norgen Biotek Corp, Ontario, Canada) and following manufacturer’s instructions. In some cases a further DNA extraction was necessary from additional paper circles.

As previously described for diagnosing *Plasmodium* species in human blood samples [13], a *Plasmodium* spp. rRNA gene fragment (ssrRNA 18S) of about 250-bp was amplified. Then a *P. falciparum*, *P. malariae*, *P. ovale*, and *Plasmodium vivax* species-specific PCR protocol was employed on all genus-specific PCR positive samples, with primers and thermal profiles described in Table 1, to amplify fragments of about 276, 412, 375, 300 bp, respectively. An amplification control based on the human beta-actin gene was performed in parallel to monitor PCR inhibition and to control for DNA integrity. PCR amplification was performed in a final volume of 25 μL under the following conditions: 10× buffer including 1.5 mM MgCl_2_, 0.2 mM of each dNTP, 1 unit of DNA polymerase (BIOTAQ™ DNA Polymerase, Aurogene, Rome, Italy) and 5 μL of template. The primer concentrations were 15 pmol of *P. falciparum*, 17.5 pmol of *P. ovale* and *P. malariae*, and 7.5 pmol of *P. vivax* forward.

**Table 1:** Primer sets and PCR conditions for DNA amplification of *Plasmodium* species

*Plasmodium* species	Primers	Product size (bp)	PCR conditions
*Plasmodium* spp.	PgMt19F3 5′-TCG CTT CTA ACG GTG AAC-3′ PgMt19B3 5′-AAT TGA TAG TAT CAG CTA TCC ATA G-3′	250	95 °C, 15′; 5 cycles [95 °C 10″, 62 °C 30″, 72 °C 15″]; 45 cycles [95 °C 10″, 62 °C 10″, 72 °C 15″] 72 °C, 10′
*P. falciparum* forward	5′-AAC AGA CGG GTA GTC ATG ATT GAG-3′	276	95 °C, 15′; 43 cycles [95 °C 45″, 60 °C 90″, 72 °C 45″]; 72 °C, 10′
*P. malariae* forward	5′-CGT TAA GAA TAA ACG CCA AGC G-3′	412	
*P. ovale* forward	5′-CTG TTC TTT GCA TTC CTT ATG C-3′	375	
*P. vivax* forward	5′-CGG CTT GGA AGT CCT TGT-3′	300	
*S*pecies-specific reverse	5′-GTA TCT GAT CGT CTT CAC TCC C-3′		

To test the specificity of the reaction, 5 μL of DNA extracted from blood samples microscopically positive for *Plasmodium* were included in each PCR run as positive control, whereas 5 μL of double-distilled water was employed as negative control. Amplicons were purified using the SureClean kit (Aurogene, Rome, Italy) following manufacturer’s instructions, and were then directly sequenced with PCR primers in both directions by an external sequencing core service (Eurofins Genomics, Anzinger, Germany). The resulting chromatograms were analysed and edited. The obtained sequences were compared to those from *Plasmodium* deposited in GenBank™ and available at the website: http://www.ncbi.nlm.nih.gov by using the BLAST application, and aligned using the software CLUSTAL X (www.clustal.org) for comparative analysis. In addition, having the MA2 detect the presence of *Babesia* spp. in several blood samples, the corresponding DNA were further amplified using the primers CRYPTO F (5′-AACCTGGTTGATCCTGCCAGT-3′) and RLB-R2 (5′-CTA AGA ATT TCA CCT CTG ACA GT-3′), which amplify a fragment of approximately 800 bp of the piroplasm 18S ribosomal RNA, using the previously described protocol [14]. Amplicons were then sequenced and aligned as above.

### Statistical analyses

Different frequency distributions were evaluated by means of the χ^2^ test, and p values <0.05 were considered significant. Cohen’s K index was calculated to evaluate the concordance between the results obtained, for each sample, by each considered diagnostic.

## Results

In the last 14 days of November 2014, a total of 306 children were screened for malaria. They were aged from 1 week to <5 years (median age = 29.7 months, ratio male/female = 1.15).

**Fig. 1:**
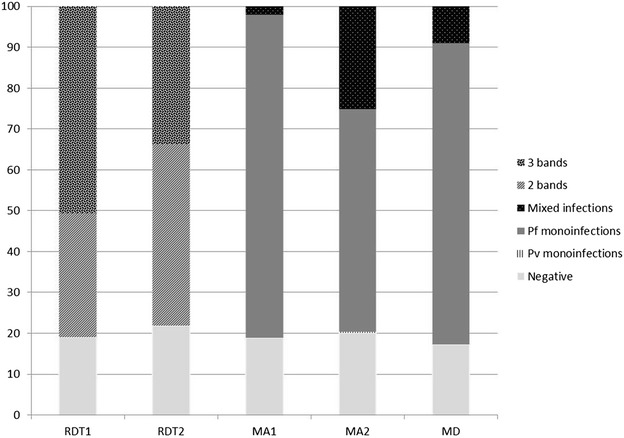
Malaria results obtained by rapid diagnostic test (RDT1 and RDT2: two-band and three-band positive), microscopic analysis (MA1 and MA2) and molecular diagnostics (MD) applied to blood samples of children living in Tshimbulu (Western Kasai, DRC). Pf = *P. falciparum*; Pv = *P. vivax*; Mixed = ≥2 *Plasmodium* species

Prevalence evidenced by each applied method is reported in Table 2 and Fig. 1, while Table 3 summarizes species identification.

**Table 2:** Prevalence of malaria and of mono-infections (1sp) detected by rapid test (RDT1 and RDT2), microscopic analysis (MA1 and MA2) and molecular diagnostics (MD) applied to children living in Tshimbulu (Western Kasai, DRC), and p values obtained by comparing the different results

Pos/ex	(%)							
	RDT1	MA1	MA1 1sp	RDT2	MA2	MA2 1sp	MD	MD 1sp
	(80.9)	(81.2)	(79.2)	(78.2)	(80.0)	(54.8)	(82.8)	(73.7)
RDT1		0.937		0.406				
238/294								
MA1	0.937				0.711		0.734	
242/298								
MA1 1sp						<0.0001		0.157
236/298								
RDT2	0.406				0.589			
222/284								
MA2		0.711		0.589			0.505	
232/290								
MA2 1sp			<0.0001					<0.0001
159/290								
MD		0.734			0.505			
164/198								
MD 1sp			0.157			<0.0001		
146/198

**Table 3:** *Plasmodium* species and their absolute (%) and relative prevalence (R %) identified by microscopic analyses (MA1 and MA2) and molecular diagnostics (MD) applied to blood samples of children living in Tshimbulu (Western Kasai, DRC) (*P.f.* = *P. falciparum; P.m.* = *P. malariae; P.o.* = *P. ovale; P.v.* = *P. vivax*)

Species	No. (%; relative %)		
	MA1	MA2	MD
No. valid for classification	298	290	198
Negative	56	58	34
*P.f.*	236 (79.2; 97.5)	158 (54.5; 68.1)	146 (73.7; 89.0)
*P.v.*	0	1 (0.3; 0.4)	0
*P.f.* + *P.m.*	0	38 (13.1; 16.4)	13 (6.6; 7.9)
*P.f* + *P.o.*	6 (2.0; 2.5)	6 (2.1; 2.6)	2 (1.0; 1.2)
*P.f.* + *P.v.*	0	8 (2.8; 3.4)	1 (0.5; 0.6)
*P.f.* + *P.m.* + *P.o.*	0	1 (0.3; 0.4)	0
*P.f.* + *P.m.* + *P.v.*	0	1 (0.3; 0.4)	1 (0.5; 0.6)
*P.f.* + *B. microti*	0	19 (6.6; 8.2)	2 (1; 1.2)

The immediate diagnosis by means of the RDT failed in 12/306 analyses (3.9 %) and 80.9 % (95 % CI 76.5–85.5) of the children were classified as positive to *P. falciparum* (37.4 and 62.6 % of which were two-band and three-band positive, respectively) (Table 2; Fig. 1, line RDT1).

The local laboratory team rectified by MA a total of four negative RDT tests into positive with very low parasitaemia due to *P. falciparum*, suspected the co-presence of *P. ovale* in six smears (2.0 %) (Tables 2, 3; Fig. 1, line MA1), and evaluated the parasitic burden valid for classification slides (n = 298), so establishing an overall malaria prevalence of 81.2 % (CI 76.8–85.6). The difference in the overall infection rate highlighted by the two diagnostic tools was not significant (p = 0.937), and K index confirmed their excellent overall concordance (K = 0.83).

The revision of the RDT results classified 5 % of the 298 available tests as unsuccessful (a picture of seven results was lost) and identified 222/284 infected children. Therefore, the overall prevalence of *P. falciparum* antigens became 78.2 % (95 % CI 73.4–83.0), a result which proved to be no statistically different from the one obtained *in loco* (p = 0.406), although this revision revealed 56.7 and 43.2 % of infected children as two-band and three-band positive, respectively (Fig. 1, line RDT2): a significantly different interpretation (p < 0.0001).

The revision by MA of available thick and thin smears (Tables 2 and 3, and Fig. 1, line MA2), classified 58/290 children as negative (20.0 %; CI 15.4–24.6) and identified 158 (54.5 %; CI 49.2–59.7) as affected by *P. falciparum*, one patient by *P. vivax* (0.3 %) and 73 (25.2 %; CI 62.9–83.1) with mixed infection, for an overall infection rate of 80.0 % (CI 75.4–84.6), not statistically different from MA1 and RDT2 results (p = 0.711 and p = 0.589, respectively). However, on a quality level, MA2 identified *P. falciparum* as the most prevalent species (79.7 %; CI 75.0–85.0), followed by *P. malariae* (13.79 %; CI 9.8–17.8), *P. vivax* (3.4 %; CI 1.4–5.4), and *P. ovale* (2.4 %; CI 0.6–4.2).

*Plasmodium falciparum* was mostly detected as single infection, whereas *P. malariae* and *P. ovale* were always combined with *P. falciparum* (in 16.4 and 2.6 % of the infections, respectively) and, in one case, as a triple infection. As for *P. vivax*, this species was identified as single (n = 1) and mixed infection (with *P. falciparum* and triple infection with *P. malariae*). Furthermore, the revision of the slides evidenced the presence of *Babesia microti* in 19 smears, always associated to *P. falciparum* and classified, by RDT, as *P. falciparum* two-band (n = 12) and three-band (n = 6) infection, and ‘invalid test’ (n = 1) (Table 3, line MA2).

The prevalence of mono-infections with *P. falciparum* (54.5 %) identified by second slide examination (MA2) was significantly different from that evidenced by MA1 (79.2 %; p < 0.0001), as confirmed by the poor overall concordance (K = 0.08) observed between the results on the available comparable specimen. As expected, MD detected the highest number of infections: 164/198 (82.8 %; CI 80.3–85.7) children proved to be positive (Tables 2 and 3, and Fig. 1, line MD). In addition, MD confirmed the MA2 identification of *P. malariae*, *P. ovale* and *P. vivax* only in 13, two, two of the 198 available dried filters, respectively, and the presence of *Babesia microti* in only two of them (Table 3, line MD). Sequencing allowed the classification of the found *P. ovale* as *Plasmodium ovale wallikeri*.

The overall prevalence of single infections with *P. falciparum* identified by MD (73.7 %) is significantly different (p < 0.0001) only from that detected by MA2 (54.5 %). However, considering the results of MA2 concerning only 164 children positive to MD, the difference in prevalence of mono-infection with *P. falciparum* is withdrawn: the concordance between the two diagnostics was excellent (K = 0.84).

**Fig. 2:**
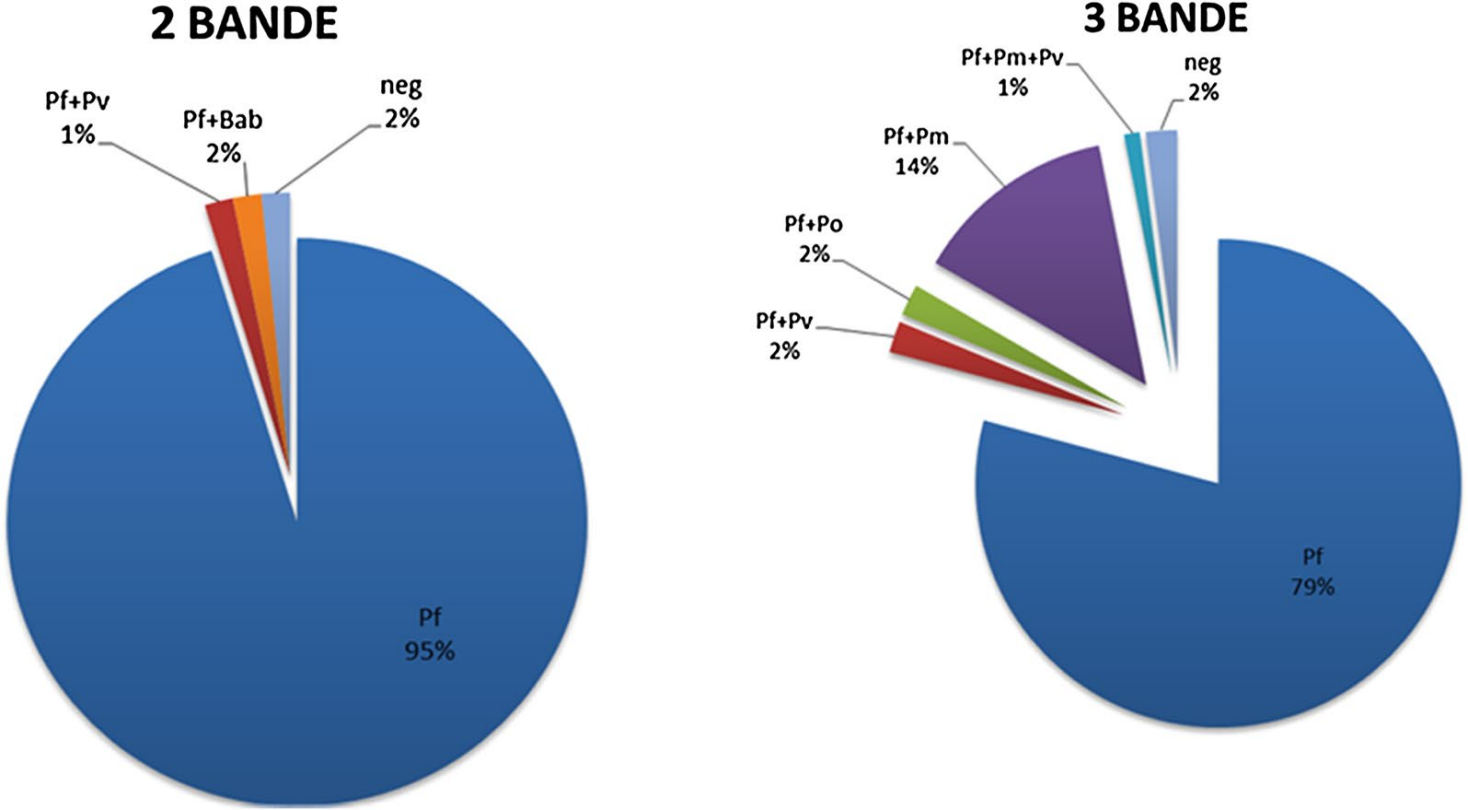
Molecular identification of *Plasmodium* species present in samples classified by RDT1 as two-band (*left*) and three-band (*right*) positive

Finally, Fig. 2 shows the molecular identification of *Plasmodium* species present in samples classified by RDT1 as two-band and three-band positive. With the exception of 4 % of the samples, which had initially been read as positive, two-band positive results were also caused by mixed infections with *P. vivax* and *Babesia microti*, whereas three-band positive by those with *P.vivax*, *P. ovale* and *P. malariae*.

With MD as the gold standard, and considering only the 198 subjects tested *in loco* with all the diagnostic tools, sensitivity and specificity of the tests *in loco* performed in detecting malaria due to *P. falciparum* were 93.1 and 92.1 % (RDT1), and 93.2 and 100 % (MA1), respectively.

## Discussion

Epidemiological data on malaria in Western Kasai, DRC, are limited and inadequate, despite this country is suffering the second-highest burden of malaria globally [2]; the infrastructure to undertake complex surveillance programmes for a reproducible and efficient surveillance are very poor. This initiative carried out on 306 children produced alarming results: more than 80 % were affected by severe malaria (as suggested by haemoglobin (Hb) measurements performed at the time of testing), and also due to the unexpected species *P. vivax*. Moreover, and for the first time in DRC, human infections with the zoonotic *Babesia microti*, which may contribute to anaemia, were detected.

Comparing the present findings with available data, malaria prevalence (82.8 %) evidenced by MD in children of Tshimbulu pointed out a sanitary problem which turned out to be more alarming (43.7 %) than reported by molecular studies on malaria epidemiology in Western Kasai [3] and, in general, in DRC (33.5 %, 34.1 %) [2, 3]. Furthermore, the presence of *P. malariae* and *P. ovale* (with relative prevalence 8.5 and 1.2 %) was confirmed and the additional presence of *P. vivax* was evidenced. In the studied area, where the lack of the Duffy antigen is widespread, the existence, although at very low prevalence (1.2 %), of this unexpected *Plasmodium* species should draw attention to the possible clinical and epidemiological consequences. Indeed, it is well known that: i) *P. vivax* gametocytes are produced earlier than those of *P. falciparum* [15], and are transmissible to *Anopheles* mosquito vectors at lower parasite densities than those of *P. falciparum*, and more efficiently [16]; therefore, transmission before diagnosis or treatment may occur [17]; ii) it is often the ‘last parasite standing’ following *P. falciparum* elimination [18, 19]; and, iii) as *P. falciparum*, this species is resistant to chloroquine [5]. That is: all the biological features of *P. vivax* make it necessary to be aware of its geographical distribution in order to plan appropriate control measures, aside from applying a suitable therapy to infected subjects.

Notwithstanding these relevant findings, the accuracy of diagnoses performed in the limited resource setting of DRC and, then in a research laboratory, was not entirely satisfactory. Indeed, considering that MD was not applied to 106 samples (93 not collected plus 13 not suitable dried spots), and, therefore, in these cases the three methodologies could not be compared, analysis of the results obtained by means of RDT, MA of the blood and MD, suggests the following observations. In respect of overall infection rates, RDT1 and MA1 results were in greater agreement with MD than RDT2 and MA2 findings that, however, were not significantly different from MD (Table 2). On the contrary, when the results are analysed in detail, only MA2 is in agreement with MD: there are four *Plasmodium* species affecting children in Tshimbulu, and *Babesia microti* is present, even if all of them are in low percentages, which means *in loco* performed analyses are satisfactory in detecting infection, but not as satisfactory as identifying the species involved.

However, each diagnostic tool used *in loco* and then, in a research laboratory during the revision of the pictures of RDT, smears and dried filter papers from Tshimbulu, confirmed the intrinsic bias, which may explain the contrasting results.

Locally applied RDT demonstrated its usefulness for rapid diagnosis of malaria; however, it is useful to note that at least 1.9 % of infections remained undiagnosed, and Pan weak bands were exposed to doubtful interpretation, probably due to partial corruption during transportation (it is known that test line intensity may decrease with either parasite density and exposure to >70 % humidity and/or >30 °C). Therefore, in respect of the performance assured (for *P. falciparum*: sensitivity 99.7 % and specificity 99.5 %), the employed RDT proved to be less sensitive and specific. Moreover, as expected (data sheet informs that three-band positive sample do not means mixed infection), it did not allow identification of mixed infections and, even less so, the species involved in infections, data very important for possible relapses due to the presence of *P. vivax* and *P. ovale* and for possible renal damage induced by *P. malariae*. Considering the additional well-known criticism about the inability to evaluate parasitic load, which is essential to define the severity of the infection, the immediate measures necessary, and the treatment efficiency, RDT cannot be the only employed method.

Regarding MA, the present findings demonstrate its high performance in detecting all *Plasmodium* species present only when carried out by well-trained microscopists in a laboratory setting. Otherwise, when a high number of children are awaiting diagnosis, important detail of infections (such as mixed infections) may be overlooked or not recognized. The lower (not significant) sensitivity shown by MA2 (80.0 %) when compared to MD (82.8 %), accepting as a true result the presence of 15 microscopically negative smears (as confirmed by negative RDT) in the first group of children unexamined by MD, becomes less relevant, and totally overlaps the results from children examined by both diagnostics (100 % sensitivity). The same applies for mixed infections: several mixed infections detected by MA2 in the first 100 children may have been lost by the unapplied MD, so that the prevalence of mono-infections with *P. falciparum* stated by MD is only apparently higher than that identified by MA2, as confirmed by the excellent concordance (K = 0.86) about simple and mixed infections observed on the remaining 164 children proved positive to both diagnostics. However, results obtained by this method sometimes proved to be uncorrected. In detail, co-infections of *P. falciparum* with *P. malariae* diagnosed during the revision of the slides proved overestimated, may be due to a misidentification of *P. falciparum* in cases of severe malaria (the data on corresponding children reported a very low Hb concentration). Indeed, due to *P. falciparum* cyto-adherence, in these cases peripheral blood included trophozoites developed further than usual little rings: they are very alike *P. malariae* and infection may be identified as co-infection. This pitfall has been overcome during MA1 because the local team, who frequently identify severe malaria, is probably used to recognizing *P. falciparum* in these conditions. The second (possibly in the absence of corresponding MD identification) misdiagnosis of MA2 (namely some *P. falciparum* interpreted as *P. vivax/ovale*) occurred in cases of macrocytic anaemia and when slides were badly stained, which make it reasonable that there was an absence of evident Schüffner’s granulations: some single infection with *P. falciparum* may have been read as possible mixed infection. Finally, *Babesia microti* has been over identified, probably as it is very similar to *P. falciparum* when just entered in the erythrocyte. In these circumstances, as well as to distinguish the two major forms of *P. ovale*, parasite morphology proved unsuitable for a systematic analysis of the relationship between the different species and only PCR-based methods proved to be reliable. However, in this study several underestimated mixed infections diagnosed by MD were observed. It could be strictly linked to the difficulties in extracting DNA from the dried blood spots and, consequently, to the possibility of identifying only the most abundant species. Therefore, MD may present weak spots that affect its performance.

As for the comparison of the performance shown by the applied malaria tests, the higher (however in this case not significant) sensitivity of MD in respect to MA and RDT, and the ~90 % specificity of the RDT used, reported in previous studies [3, 20] were confirmed. However, in this experience it is crucial to underline the ability of the local team in detecting positive slides (81.2 % instead of 82.8 %), higher than that reported in the abovementioned paper (22.7 *vs* 34.1 %) [20].

## Conclusions

The findings in Tshimbulu pointed out worrying malaria prevalence in children, mostly without symptoms of the disease, due to four *Plasmodium* species. In addition, the zoonotic *Babesia microti*, which may contribute to their anaemia, was detected.

As for the accuracy of *in loco* applied diagnostic tools, if a rapid replay of a clinical suspicion is very useful, the importance of both easy interpretation of the test and the identification of mixed infections cannot be underestimated. The RDT used, like most RDT, is exposed to doubtful interpretation and is unable to offer important detail of an infection, therefore, the production of devices that recognize all species, *P. knowlesi* included, and characterize mixed infections should be promoted.

This experience highlighted a satisfactory level of competence in a rural hospital laboratory team in evidencing positive children, but several difficulties in identifying *Plasmodium* species, which is a major issue for all microscopists in any diagnostic setting. This difficulty may be overcome by improving the current practice of microscopy through the use of available on-loan sets of thick and thin malaria smears, self-testing for competency in malaria microscopy [21], and newer and more sophisticated assays and technical platforms, such as that used by the local team: a m-phone practical training [22].

## Abbreviations

DRC: Democratic Republic of the Congo
RDT: rapid diagnostic test
MA: microscopic analysis
MD: molecular diagnostics
